# Optimization of Atmospheric Pressure Plasma Jet with Single-Pin Electrode Configuration and Its Application in Polyaniline Thin Film Growth

**DOI:** 10.3390/polym14081535

**Published:** 2022-04-10

**Authors:** Eun Young Jung, Choon-Sang Park, Hyo Jun Jang, Shahzad Iqbal, Tae Eun Hong, Bhum Jae Shin, Muhan Choi, Heung-Sik Tae

**Affiliations:** 1 School of Electronic and Electrical Engineering, College of IT Engineering, Kyungpook National University, Daegu 41566, Korea; eyjung@knu.ac.kr (E.Y.J.); bs00201@knu.ac.kr (H.J.J.); shahzadiqbal@knu.ac.kr (S.I.); mhchoi@ee.knu.ac.kr (M.C.); 2 Department of Electrical Engineering, Milligan University, Johnson City, TN 37682, USA; cpark@milligan.edu; 3 Division of High-Technology Materials Research, Korea Basic Science Institute, Busan 46742, Korea; tehong@kbsi.re.kr; 4 Department of Electronics Engineering, Sejong University, Seoul 05006, Korea; hahusbj@sejong.ac.kr; 5 Digital Technology Research Center, Kyungpook National University, Daegu 41566, Korea; 6 School of Electronics Engineering, College of IT Engineering, Kyungpook National University, Daegu 41566, Korea

**Keywords:** atmospheric pressure plasmas, glow-like discharge, single pin electrode, plasma deposition, PANI thin film

## Abstract

This study systematically investigated an atmospheric pressure plasma reactor with a centered single pin electrode inside a dielectric tube for depositing the polyaniline (PANI) thin film based on the experimental case studies relative to variations in pin electrode configurations (cases I, II, and III), bluff-body heights, and argon (Ar) gas flow rates. In these cases, the intensified charge-coupled device and optical emission spectroscopy were analyzed to investigate the factors affecting intensive glow-like plasma generation for deposition with a large area. Compared to case I, the intense glow-like plasma of the cases II and III generated abundant reactive nitrogen species (RNSs) and excited argon radical species for fragmentation and recombination of PANI. In case III, the film thickness and deposition rate of the PANI thin film were about 450 nm and 7.5 nm/min, respectively. This increase may imply that the increase in the excited radical species contributes to the fragmentation and recombination due to the increase in RNSs and excited argon radicals during the atmospheric pressure (AP) plasma polymerization to obtain the PANI thin film. This intense glow-like plasma generated broadly by the AP plasma reactor can uniformly deposit the PANI thin film, which is confirmed by field emission-scanning electron microscopy and Fourier transform infrared spectroscopy.

## 1. Introduction

In recent decades, the atmospheric pressure plasma (APP) process has attracted much attention due to its many advantages, such as low cost and fast operation, low temperature, operation in air, and the ability to produce reactive chemistry at room temperature [1,2]. Thus, the APP process has enabled technology in several biological and industrial applications, such as thin-film deposition, nanomaterial synthesis, polymeric surface modification, and biomedical applications [1,2,3,4,5,6]. Numerous research groups have developed various kinds of plasma devices based on methods of plasma generation and studied using the discharge plasma based on different geometries using various electrode materials [1,7,8,9,10,11,12,13,14,15].

Our group has proposed a new plasma polymerization technique adopting an additional glass-tube and bluff-body system. Additionally, we have been researching the synthesis of polymers and copolymers using AP plasma processing [16]. Recently, a new AP plasma reactor (APPR) with a needle electrode has been proposed by J.Y. Kim et. al. [17,18]. However, no detailed experimental results exist for various electrode configurations in atmospheric plasma. Therefore, it is necessary to specifically investigate case studies on the various electrode configurations for a high deposition rate to overcome the localized area deposition. In particular, we focused on the various case studies of pin electrode configurations for overcoming the localized area deposition problem by supplying excited species formed within the gas-feeding tube into the nucleation (or fragmentation) region.

Accordingly, we systematically investigate the plasma properties of APPR with three pin electrode configurations, argon flow rates, and bluff-body heights. The plasma characteristics of APPR are investigated using an intensified charge-coupled device (ICCD) and optical emission spectroscopy (OES). Moreover, we investigated the characteristics of the deposited PANI thin film prepared by APPR with respect to three electrode configurations. The deposited polyaniline (PANI) thin films were characterized using field emission-scanning electron microscopy (FE-SEM), atomic force microscopy (AFM), stylus profiler, and Fourier transform infrared spectroscopy (FTIR).

## 2. Materials and Methods

### 2.1. Experimental Setup

**Figure 1:**
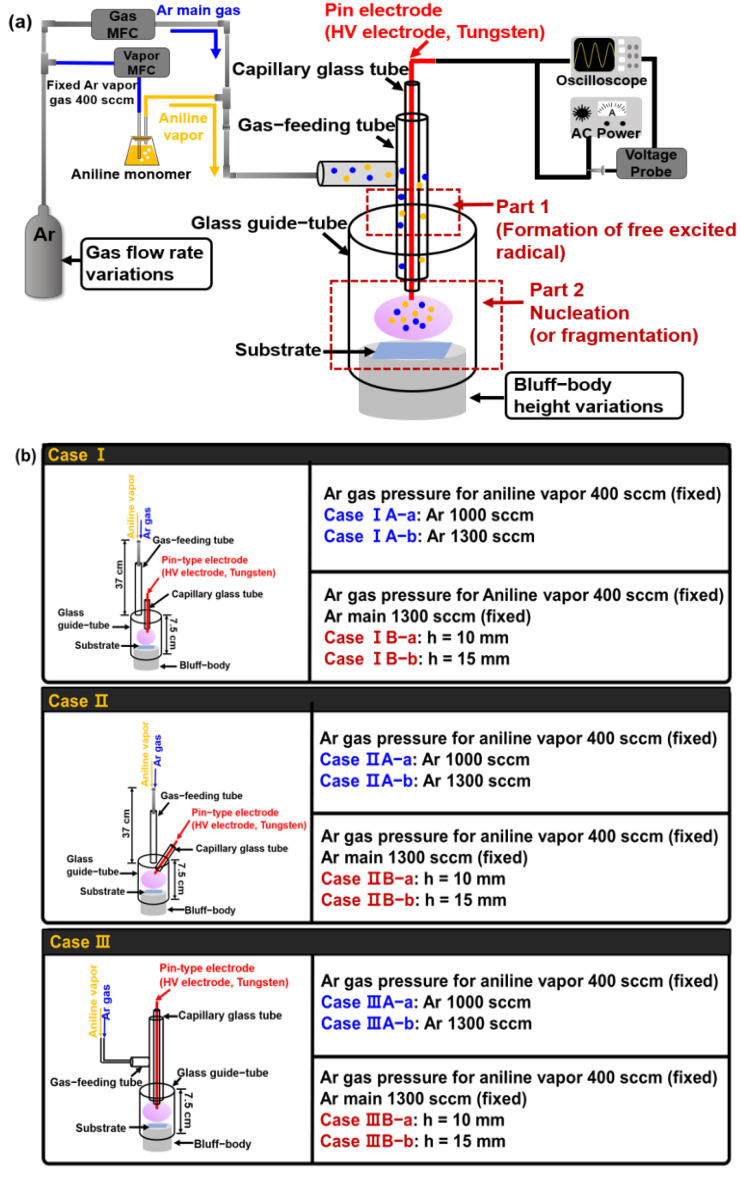
(**a**) Experimental setup, (**b**) schematic for three electrode configurations of APPR, and the detailed case studies used herein.

Figure 1 shows the experimental setup and the configuration of the APPR used. Herein, we mainly focused on the three pin electrode configurations (cases I, II, and III) to generate glow-like discharge for depositing a large-area polymer thin film. For all cases, the APPR comprises a glass tube for feeding the gas (i.e., gas-feeding tube), a glass guide-tube for generating plasma, a bluff-body, a capillary glass tube, and a centered pin electrode made up of a 0.5 mm diameter tungsten needle. The tungsten wire electrode was covered with a capillary glass tube, and the tip of the tungsten wire was protruded at 2 mm from the end of the capillary glass tube. Additionally, the glass guide-tube has a length and an outer diameter of 7.5 cm and 34 mm, respectively. The gas injection glass tube has a length and inner diameter of 37 cm and 6.8 mm, respectively. The bluff-body was made of polytetrafluoroethylene insulating material, and the substrate was placed on the bluff-body inside the glass guide-tube. The bluff-body position with respect to the guide-tube significantly influences the production of intense and broadened plasma in the nucleation (or fragmentation) region.

Moreover, this APPR was used with a single pin electrode configuration with no grounded electrode. In particular, in case I, the pin electrode was vertically placed in the center of a glass guide-tube parallel to the gas-feeding tube. In case II, the pin electrode was titled at ~50° on the side of the glass guide-tube and was also separated from the gas-feeding tube. In case III, the pin electrode was vertically combined into the gas-feeding tube on top of the glass guide-tube in the APPR to form excited radical spaces at the gas-feeding tube for large-area plasma expansion. The case III structure is largely divided into two parts. Part 1 forms the free excited radical for injecting the excited radical spaces in the gas-feeding tube. Part 2 is the region where the nucleation (or fragmentation) reaction occurs by the injected free excited radical species for polymer thin-film deposition.

The aniline monomer solution was coated on glass and silicon wafer substrates by the proposed APPR with various pin electrodes. Accordingly, to produce the intense glow-like plasma, three case studies were systematically investigated using three electrode configurations, different argon flow rates, and bluff-body heights. The detailed case studies for generating plasma discharge are presented in Figure 1 and Table 1.

High-of argon (99.999%) was used as the main gas for producing the intense glow-like plasma. Aniline monomer (MW = 93 g∙mol^−1^, Sigma-Aldrich Co., St. Louis, MO, USA) solution was vaporized using a glass bubbler with a 40-mL amount, which was supplied by argon flowing at 400 standard cubic centimeters per minute (sccm). A bipolar sinusoidal voltage wave pulse with a peak-to-peak (V _p-p_) value of 8 kV and a frequency of 30 kHz was applied to the powdered pin electrode to produce the plasma. The characteristics of the plasma produced by the APPR strongly depend on the system configuration, gas flow rate, and bluff-body height.

### 2.2. Intensified Charge-Coupled Device (ICCD)

The generated plasma investigations were conducted using an ICCD camera (PI-MAX 2, Princeton Instruments, Trenton, NJ, USA) in both shutter modes with 100 ms exposure time to identify the spatial distribution of the generated glow plasma.

### 2.3. Discharge Voltage and Current Waveform Analysis

The applied voltage and discharge current were obtained using a high-voltage probe (P6015A, Tektronix Inc., Beaverton, OR, USA) and a current monitor (Pearson 4100, Pearson Electronics Inc., Palo Alto, CA, USA), respectively. The electrical signals were monitored and stored through a digital oscilloscope (WaveRunner 64Xi, Teledyne LeCroy Inc., Chestnut Ridge, NY, USA). The discharge current was obtained by subtracting the current waveform obtained when the plasma was turned off by stopping the argon supply from the current waveform, measured when the plasma was turned on.

### 2.4. Optical Emission Spectroscopy

To investigate the excited radical species present in the generated plasma discharge due to the interaction between the aniline monomer and argon plasma, OES techniques were used to measure and analyze the optical intensities and spectra of the excited nitrogen and argon peaks, using a fiber optic spectrometer (Ocean Optics Inc., USB-4000, Dunedin, FL, USA) associated with a 1 mm diameter optical fiber and a collimating lens. The spectral resolution of the instrument was 0.06 nm.

### 2.5. Field Emission-Scanning Electron Microscopy

The surface morphology images of the PANI thin films were examined using FE-SEM (Hitachi SU8220, Hitachi High-Technologies, Tokyo, Japan) with accelerated electrons at a voltage and current of 3 kV and 10 μA, respectively. The samples for FE-SEM were made conductive by coating them with platinum before loading into the chamber.

### 2.6. Stylus Profiler

The film thicknesses of the PANI thin films were obtained using a stylus profiler (KLA Tencor, P-7, KLA Tencor Corp., Milpitas, CA, USA) at the Korea Basic Science Institute (KBSI; Busan, Korea). Measurements were performed while moving the stylus in contact with the PANI film surface at a scan speed of 200 μm/s.

### 2.7. Atomic Force Microscopy

The surface roughness characteristics of the PANI thin films were investigated in a on a noncontact mode by AFM (NanoWizard II, Brucker, Berlin, Germany) at the Korea Basic Science Institute (KBSI; Busan, Korea). All measurements were obtained under controlled room temperature. Moreover, the scanning area was 20 μm × 20 μm, and the scan rate was set at 1 Hz. Bruker NanoWizard software was used for image processing and interpretation.

### 2.8. Fourier Transform Infrared Spectroscopy

The main functional groups and crystalline phases of the PANI thin films were measured by FTIR (Vertex 70, Bruker, Ettlingen, Germany) at the KBSI (Daegu, Korea). The FTIR spectra were measured by averaging 128 scans at a wavenumber resolution of 0.6 cm^−1^ ranging from 650–4000 cm^−1^ in the attenuated total reflection (ATR) mode.

## 3. Results

**Figure 2:**
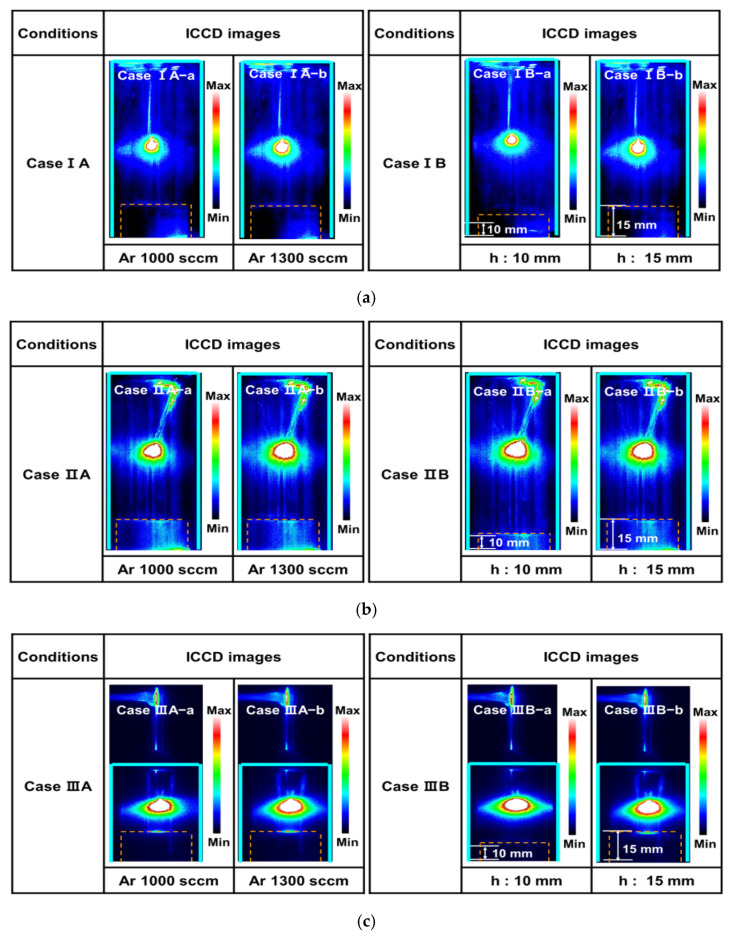
ICCD images of plasma produced in the APPR with pin electrode with respect to three different electrode configurations of cases (**a**) I, (**b**) II, and (**c**) III.

Figure 2 shows the ICCD images of plasma discharge generated by the proposed APPR with various case studies such as three electrode configurations, argon flow rates, and bluff-body heights. To optimize the geometry of the proposed APPR for large-area deposition, we investigated the various cases, namely, three different electrode configurations (cases I–III), two different gas flow rates, and two different bluff-body heights inside the guide-tube. The detailed case studies for generating plasma discharge are presented in Figure 1 and Figure 2.

For all three cases, the plasma intensity increased when the main gas (argon) flow rate increased from 1000 to 1300 sccm (Figure 2). This result implies that the higher argon flow is essential in generating the glow discharge with highly intense cloud-like glow plasma [19,20,21,22]. Additionally, for all cases, when changing the bluff-body height from 10 (cases IB-a, IIB-a, and IIIB-a) to 15 mm (cases IB-b, IIB-b, and IIIB-b), with other conditions kept constant, the produced plasma discharge was highly intense.

Moreover, the ICCD images show that the intense plasma is generated at the vicinity of the needle electrode applied by high voltage [23,24,25,26]. In particular, the produced plasma in case III was spatially expanded into the horizontal space inside the guide-tube of the APPR due to the nucleation and fragmentation reactions through the injected free excited radical species. These results confirmed that optimal conditions were required to generate the glow discharge with intense glow-like plasma for PANI thin film deposition. Based on the experimental results of Figure 2, the optimal conditions were obtained for producing highly intense plasma and synthesizing PANI thin film. The optimal conditions are argon flow rate and bluff-body height of 1300 sccm and 15 mm, respectively.

**Figure 3:**
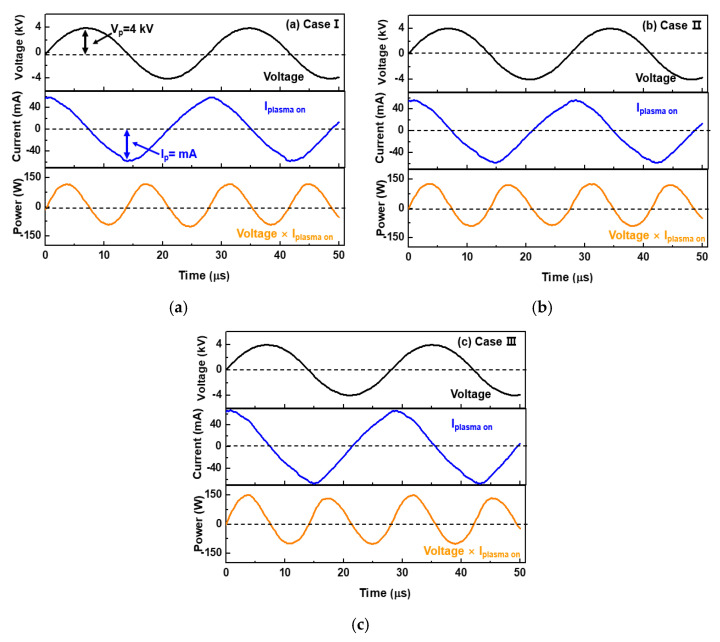
Characteristics of applied voltage, total current in plasma ON state, and instantaneous power of the APPR under optimal conditions (argon flow rate = 1300 sccm and bluff − body height = 15 mm) with respect to three electrode configurations of cases (**a**) I, (**b**) II, and (**c**) III.

To identify the discharge behavior of the APPR at an optimal condition for the three-pin electrode configurations (cases I, II, and III), the applied voltage, total current in plasma ON state, and instantaneous power were each measured as a function of time, and the results are indicated in Figure 3.

For all cases, the average power, P_a_ in the APPR was calculated from Equation (1).

(1) $$P_{a}=\frac{1}{T}\int_{0}^{T}V\left(t\right)\times I\left(t\right)\mathrm{dt}$$

where T is the applied voltage period, V(t) is the voltage signal, I(t) is the acquired current, and t is the time. The average power during 1 period was calculated through the integrated value of the power waveform during 1 period. Consequently, the average power values of the plasma reactors in cases I, II, and III are 0.8, 1.5, and 1.6 W, respectively. Table 2 summarizes the detailed experimental results of the APPR under optimal conditions with three electrode configurations. Thus, it was confirmed that case III exhibited the highest dissipated power, mainly because excited radical species were produced within the gas-feeding tube.

**Figure 4:**
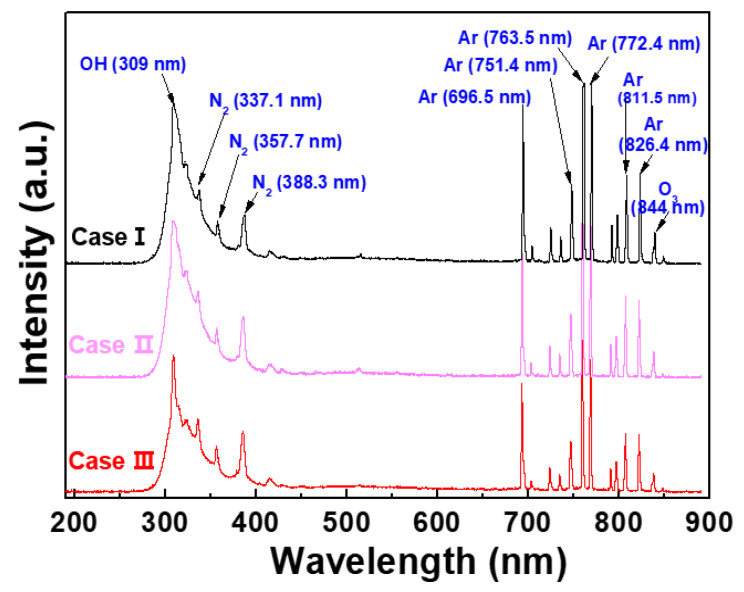
OES spectra of APPR under optimal conditions with respect to three electrode configurations of cases I, II, and III.

To investigate the mechanism of the produced plasma with an intense glow-like discharge and the effect of the excited reactive radical species produced by the APPR with a single-pin electrode using argon discharge, OES measurements were conducted to investigate the excited reactive radical species, such as nitrogen, oxygen, and argon radical species in the APPR with single-pin electrode for three different electrode configurations (cases I, II, and III) under optimal conditions. Figure 4 shows the OES spectra measured in the plasma plumes of the APPR. Consequently, several peaks of excited nitrogen (N_2_; 337.1, 357.7, and 388 nm), oxygen (OH radicals), and argon peaks were observed at the wavelength ranging from 300–900 nm [19,20,21,22]. In particular, when compared to case I, case III’s peak intensities of the excited N_2_ and Ar radical species increased, resulting from a frequent collision reaction between gas mixtures. Herein, these N_2_ peaks indicate a higher concentration of reactive nitrogen species (RNSs), which are essential in depositing PANI polymer films [16]. Based on these results, the increase in excited radical species in case III could contribute to nucleation and fragmentation reactions through the injected free excited radical species for depositing PANI thin films.

**Figure 5:**
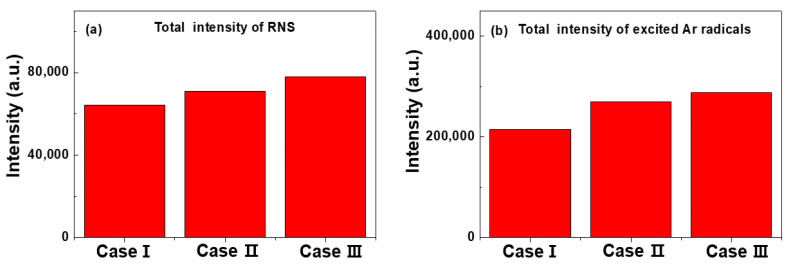
Total peak intensity of (**a**) excited RNS and (**b**) argon radicals from OES spectra under optimal conditions with respect to three electrode configurations of cases I, II, and III.

To compare the OES analysis quantitatively among the RNS and excited argon radicals, the total emission intensities were calculated from each emission intensity. Figure 5a,b show the total peak intensities of RNS and excited argon radicals calculated from the OES spectra of Figure 4, respectively. The total peak intensities of RNS in Figure 5a,b are the sum of the peak intensities of several RNS obtained from the OES spectra of Figure 4, where the wavelengths of several RNS are 337.1, 357.7 and 388.3 nm. The total peak intensities of exited argon radicals in Figure 5b are the sum of the peak intensities of several exited argon radicals obtained from the OES spectra of Figure 4, where the wavelengths of several exited argon radicals are 696.5, 751.4, 763.5, 772.4, 811.5 and 826.4 nm. The increase in the total peak intensities of RNS and excited argon radicals is related to the argon flow rate, including the bluff-body position in which the substrate is placed. In particular, in case III, it was confirmed that the higher density plasma could expand in the horizontal direction inside the glass guided-tube due to the formation of excited radical spaces within the gas-feeding tube. Based on the experimental results of Figure 2, Figure 3, Figure 4 and Figure 5, the optimal deposition conditions were chosen for depositing the PANI thin films.

**Figure 6:**
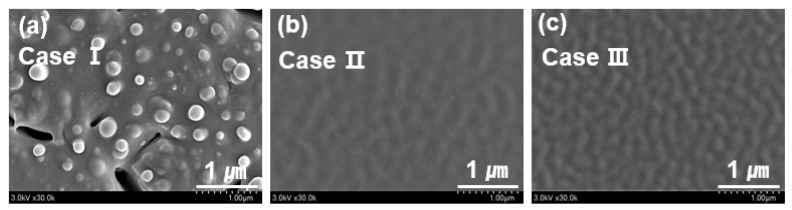
FE-SEM images of PANI thin films prepared using APPR with pin electrode under optimal conditions with respect to three electrode configurations of (**a**) cases I, (**b**) II, and (**c**) III.

The morphologies of the deposited PANI thin films were investigated under the optimal conditions with respect to three electrode configurations. Figure 6 shows the SEM images of the deposited PANI thin films under the optimal conditions using the APPR with respect to three electrode configurations. For case I, spherical particles were observed on the surface of the deposited PANI thin film (Figure 6a). However, for cases II and III, the surface of the deposited PANI thin film was homogeneous and smooth (Figure 6b,c). Thus, these results show that the surface morphologies of PANI thin films are strongly affected by the pin electrode configurations of APPR.

**Figure 7:**
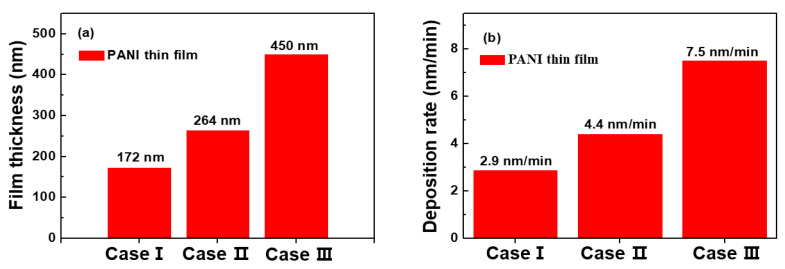
(**a**) Film thickness and (**b**) deposition rate of PANI thin film prepared by APPR with pin electrode under optimal conditions when using three electrode configurations (cases I, II, and III).

Figure 7a,b show the film thicknesses and deposition rate variations for PANI thin films deposited by the APPR with the pin electrode on glass substrates under optimal conditions for 1 h with respect to three electrode configurations. For case III, the film thickness and deposition rate of the PANI thin film were about 450 nm and 7.5 nm/min, respectively. For case III, the PANI thin film with the highest film thickness and deposition rate was obtained. This trend may be mainly due to the supply of excited species, such as RNS and argon radicals, formed within the gas-feeding tube into the nucleation or fragmentation region. The additional increase in RNS and argon radicals within the gas-feeding tube requires additional power consumption, which is confirmed by a higher dissipated power during AP polymerization for case III (Table 2).

**Figure 8:**
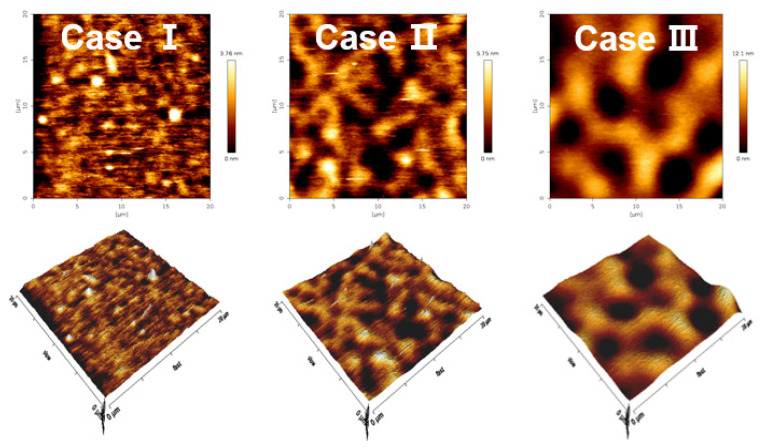
Two- and three-dimensional AFM images of PANI thin films prepared by APPR with pin electrode under optimal conditions with respect to three electrode configurations (cases I, II, and III).

Figure 8 shows the changes in two- and three-dimensional AFM images of PANI thin films deposited on glass substrates for 1 h. The root means’ square roughness (R_rms_) and average roughness (R_a_) obtained from the AFM images of PANI thin film surfaces of Figure 8 are summarized in Table 3. First, for case I, the surface R_a_ and R_rms_ values are 0.22 and 0.75 nm, respectively. Second, for case II, the surface R_a_ and R_rms_ values are 1.03 and 1.31 nm, respectively. Finally, for case III, the surface R_a_ and R_rms_ are 0.61 and 0.85 nm, respectively. Thus, these results show that the surface roughness characteristics of the PANI thin films are significantly affected by the pin electrode configurations of the APPR.

**Figure 9:**
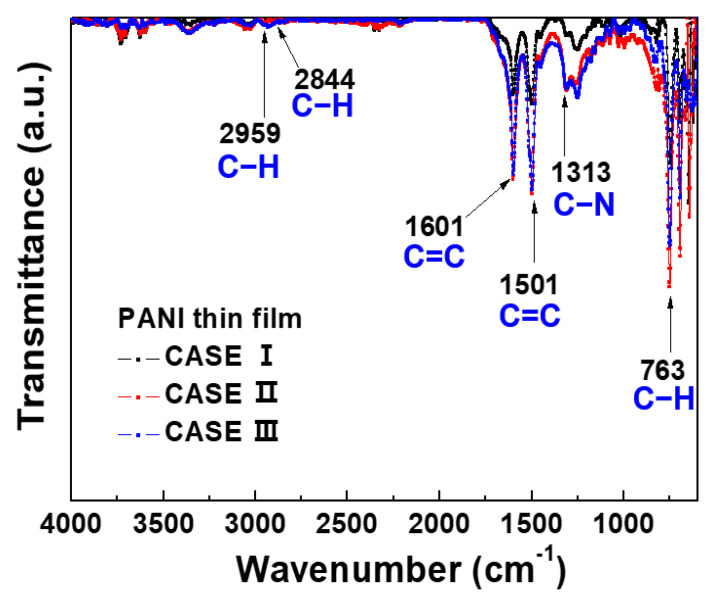
FTIR spectra of PANI thin films prepared by APPR with pin electrode under optimal conditions with respect to three electrode configurations (cases I, II, and III).

Figure 9 shows the FTIR absorption spectrum of the PANI thin films deposited on silicon wafer substrates for 1 h by APPR under optimal conditions with respect to three electrode configurations (cases I, II, and III). Herein, all spectra show the characteristic peaks of the PANI polymer at 2959, 2844, 1601, 1501, 1313, 1250, and 763 cm^−1^. The peaks at 1501 and 1601 cm^−1^ are attributed to benzenoid and quinoid ring stretching vibrations, respectively. The band at 763 cm^−1^ is ascribed to the C–H out-of-plane deformation from the aromatic ring, and the peak at 1313 cm^−1^ is attributed to the C–N stretching vibration [17,27]. In addition, the assigned peaks at 2844 and 2959 cm^−1^ are attributed to the stretching within the polymer chains [17,27]. From the FTIR spectra, the peak assignments of the PANI thin films deposited by APPR are represented in Table 4. In particular, the FTIR peak intensities of the π-conjugated bonds (1501 and 1601 cm^−1^) and the C–N bond (1313 cm^−1^) increased for cases (cases II) when compared to case I. The enhancement of the C–N peak is related to the electrical conductivity due to the nitrogen atom of the quinone ring [28,29]. Moreover, the increase in π-conjugated bonds is expected to enhance the π–π stacking of intermolecular polymer chains, thereby resulting in good carrier mobility and improved electrical conductivity [28,29]. Hence, the proposed APPR with pin electrode configuration (case III) can inject the excited radical species formed within the gas-feeding tube into the nucleation or fragmentation region. The PANI thin film grown in the proposed APPR exhibited the highest film thickness, deposition rate, and lowest roughness. Thus, the proposed APPR device is applicable to various PANI-based gas sensors [16] by overcoming the low deposition rate of conventional PANI films.

## 4. Conclusions

To broadly generate a glow-like intense plasma for depositing PANI thin films, we evaluated various case studies in detail, depending on the variations of the APPR, such as pin electrode configurations (cases I, II, and III), bluff-body heights, and argon flow rates. The morphologies, structures, and deposition rates of the PANI thin films strongly depend on pin electrode configurations. For case III, the PANI thin films show the maximum film thickness (about 450 nm) and the highest deposition rate (7.5 nm/min). The PANI thin films show homogeneous, flat, and smooth surfaces with roughness characteristics below a few nanometers, as revealed by SEM and AFM. The PANI thin films show the structural feature increasing the π-conjugated bonds (1501 and 1601 cm^−1^) and C–N bond (1313 cm^−1^), as confirmed by FTIR. This growth of high-quality PANI thin films is due to the increased excited radical species contributing to the fragmentation and recombination for obtaining the PANI thin film during plasma deposition. Therefore, it is expected that the APPR with the pin electrode in case III deposits large-area PANI thin films with a high deposition rate by overcoming the localized area deposition problem of conventional AP plasma devices.

## Figures and Tables

**Table 1:** Detailed case studies for generating the intense glow-like discharge using APPR with pin electrode used herein.

Electrode Configuration	Case I: Vertically Parallel Pin Electrode
	Case II: Titled Pin Electrode
	Case III: Vertically Combined Pin Electrode
Precursor liquid solution	Aniline monomer
Driving power source	AC sinusoidal
Plasma driving voltage (V _p-p_)	8 kV (Fixed)
Frequency	30 kHz (Fixed)
Argon pressure for aniline vapor	400 sccm (Fixed)
Argon main gas pressure	1000 sccm and 1300 sccm (controllable)
Bluff-body height	10 mm and 15 mm (controllable)

**Table 2:** Summary of experimental results of the applied voltage and average power during plasma polymerization in APPR with three-pin electrodes used herein.

Electrode Configuration	Case I	Case II	Case III
Driving type	AC	AC	AC
Voltage waveform	Sinusoidal	Sinusoidal	Sinusoidal
Plasm a driving voltage (V _p-p_)	8 kV	8 kV	8 kV
Average power	0.8 W	1.5 W	1.6 W

**Table 3:** Root mean square roughness (R_rms_) and average roughness (R_a_) of PANI thin films obtained from AFM images in Figure 8.

Sample Conditions	Case I	Case II	Case III
R_a_	2.22 nm	1.03 nm	0.61 nm
R_rms_	2.75 nm	1.31 nm	0.85 nm

**Table 4:** Comparison of FTIR spectra of PANI thin films deposited by APPR with pin electrode under optimal conditions with respect to three electrode configurations (cases I, II, and III) obtained from FTIR spectra of PANI thin films in Figure 9.

Wavenumber	Peak Assignment
763 cm^−1^	C–H out-of-plane bending
1313 cm^−1^	C–N stretching vibration
1501 cm^−1^	C=C stretching vibrations of the benzenoid rings
1601 cm^−1^	C=C stretching vibrations of quinoid rings
2844 cm^−1^	C–H stretching vibration
2959 cm^−1^	C–H stretching vibration

## Data Availability

Not applicable.
